# The integration of single-cell and bulk RNA-seq atlas reveals ERS-mediated acinar cell damage in acute pancreatitis

**DOI:** 10.1186/s12967-024-05156-0

**Published:** 2024-04-11

**Authors:** Kaige Yang, Rongli Xie, Guohui Xiao, Zhifeng Zhao, Min Ding, Tingyu Lin, Yiu Sing Tsang, Ying Chen, Dan Xu, Jian Fei

**Affiliations:** 1 Department of General Surgery, Pancreatic Disease Center, Ruijin Hospital, Shanghai Jiao Tong University School of Medicine, Shanghai, China; 2 Department of General Surgery, Ruijin Hospital LuWan Branch, Shanghai Jiaotong University School of Medicine, Shanghai, China; 3 Institute of Translational Medicine, Shanghai Jiao Tong University, Shanghai, China; 4 Department of Emergency, Ruijin Hospital, Shanghai Jiao Tong University School of Medicine, Shanghai, China; 5 Department of Gastrointestinal Surgery, Xijing Hospital, Fourth Military Medical University, Xi’an, China

**Keywords:** Single-cell RNA sequencing, Bulk RNA sequencing, Vesicle transport, ERS, ERAD, Acute pancreatitis

## Abstract

**Background:**

Acute pancreatitis (AP) is a clinically common acute abdominal disease, whose pathogenesis remains unclear. The severe patients usually have multiple complications and lack specific drugs, leading to a high mortality and poor outcome. Acinar cells are recognized as the initial site of AP. However, there are no precise single-cell transcriptomic profiles to decipher the landscape of acinar cells during AP, which are the missing pieces of jigsaw we aimed to complete in this study.

**Methods:**

A single-cell sequencing dataset was used to identify the cell types in pancreas of AP mice and to depict the transcriptomic maps in acinar cells. The pathways’ activities were evaluated by gene sets enrichment analysis (GSEA) and single-cell gene sets variation analysis (GSVA). Pseudotime analysis was performed to describe the development trajectories of acinar cells. We also constructed the protein–protein interaction (PPI) network and identified the hub genes. Another independent single-cell sequencing dataset of pancreas samples from AP mice and a bulk RNA sequencing dataset of peripheral blood samples from AP patients were also analyzed.

**Results:**

In this study, we identified genetic markers of each cell type in the pancreas of AP mice based on single-cell sequencing datasets and analyzed the transcription changes in acinar cells. We found that acinar cells featured acinar-ductal metaplasia (ADM), as well as increased endocytosis and vesicle transport activity during AP. Notably, the endoplasmic reticulum stress (ERS) and ER-associated degradation (ERAD) pathways activated by accumulation of unfolded/misfolded proteins in acinar cells could be pivotal for the development of AP.

**Conclusion:**

We deciphered the distinct roadmap of acinar cells in the early stage of AP at single-cell level. ERS and ERAD pathways are crucially important for acinar homeostasis and the pathogenesis of AP.

**Supplementary Information:**

The online version contains supplementary material available at 10.1186/s12967-024-05156-0.

## Introduction

Acute pancreatitis (AP) is a common and potentially fatal gastrointestinal disorder in the emergency department. The worldwide incidence of this disease is about 23–136/100,000 and keeps rising steadily in most Western countries [1]. In the United States, there are over 300,000 emergency cases caused by AP annually, with $9.3 billion expenses for health care [2, 3]. Abdominal pain is the most typical manifestation, possibly accompanied by nausea, vomiting and fever. About 20% of patients deteriorate to severe acute pancreatitis (SAP) featured by multiple organ failure, the mortality rate of which is up to 40% [4]. The current therapies, including early fluid resuscitation, nutritional support, anti-infection and organ function support, are mainly symptom-based and we still lack specific treatments. The complicated pathogenesis of AP remains unelucidated, although multiple hypotheses have been proposed, such as premature trypsinogen activation, aberrant calcium signaling, endoplasmic reticulum stress and impaired autophagy [3]. This study focused on demonstrating pathological events of pancreatic acinar cells in the early stage of AP, through analysis of the single-cell sequencing data of pancreas from mouse model combined the bulk RNA sequencing data of peripheral blood from AP patients.

## Methods

### Dataset accession

The single-cell RNA sequencing (scRNA-seq) datasets GSE181276 and GSE188819 from mouse pancreas samples and the bulk RNA sequencing (bulk RNA-seq) dataset GSE194331 from human peripheral blood were downloaded from the GEO database (https://www.ncbi.nlm.nih.gov/geo/). We first analyzed the wildtype (WT, untreated/control) sample GSM5494073 and the AP sample GSM5494074 from GSE181276 to identify cell types in the mouse pancreas and investigate the transcriptional changes in acinar cells during AP. Then, the results were compared with another independent analysis of two WT samples and two AP samples from GSE188819. Finally, by combining the data from peripheral blood samples from 32 healthy volunteers and 10 SAP patients from GSE194331, we identified the biological features of AP.

### Animal model

As described by respective data uploader, for the dataset GSE181276, AP mouse model was induced by 8 hourly intraperitoneal injections of cerulein (75 μg/kg for the first 3 injections, 50 μg/kg for the last 5 injections) over two consecutive days [5]. For the dataset GSE188819, AP mouse model was induced by 8 hourly intraperitoneal injections of cerulein (100 μg/kg) over two consecutive days [6]. WT mice were injected with the same dose of saline at the same time. Pancreatic tissues from representative mice were harvested the day after the last injection and dissociated into a single-cell suspension for the subsequent scRNA-seq.

### Preparation of single-cell suspension for scRNA-seq

For the dataset GSE181276, the pancreatic tissues were digested with the collagenase IV-dispase II (2 mg/ml) at 37 °C for 45 min and further treated with 0.25% trypsin solution at 37 °C for 5 min to obtain single-cell suspension [5]. For the dataset GSE188819, the pancreatic tissues were incubated with the mixture of digestive enzymes (1 mg/ml collagenase P, 2 U/ml dispase II, 0.1 mg/ml soybean trypsin inhibitor and 0.1 mg/ml DNase I in HBSS with Ca^2+^/Mg^2+^) and digested by a gentle MACS™ Octo Dissociator at 37 °C for 40 min, followed by further treatment with 0.05% Trypsin–EDTA at 37 °C for 5 min and removal of red blood cells [6].

### Pre-processing and quality control of scRNA-seq data

The scRNA-seq data of individual samples were loaded onto Seurat R package (version 4.3.0). Cells of low quality with < 200 or > 8000 total expressed genes and > 20% mitochondrial genes were filtered out. The potential doublets (paired cells) were also discarded with the DoubletFinder package (version 2.0.3) [7]. For the dataset GSE181276, there were a total of 15,819 cells retained for further analysis, including 5732 cells from WT sample and 10,087 cells from AP sample. And for the dataset GSE188819, 8381 cells from WT samples and 18998 cells from AP samples were reserved. Then the datasets GSE181276 and GSE188819 were separately analyzed.

### Data integration and dimensionality reduction

The filtered data from individual samples were merged into one Seurat object. We normalized the gene expression matrix by “LogNormalize” method and identified the top 3000 highly variable genes (HVGs). The normalized expression matrix of HVGs were centered and scaled before we conducted dimensionality reduction by the principal component analysis (PCA). Based on the top 50 PCA components, the Harmony R package (version 0.1.1) was used to remove the batch effects [8].

### Cell-clustering and annotation

We computed a shared nearest-neighbor graph and identified cell clusters based on the harmony reduction. The 2D visualization map of identified clusters was produced with uniform manifold approximation and projection (UMAP) methods. To further annotate these clusters, we identified the differentially expressed genes (DEGs) of each cluster using the “FindAllMarkers” function in Seurat with default Wilcoxon rank sum test. The cells were annotated according to DEGs and cellular markers from the CellMarker database 2.0 (http://bio-bigdata.hrbmu.edu.cn/CellMarker/index.html).

### DEGs identification and enrichment analysis

The DEGs of acinar cells between WT and AP groups were identified by the “FindMarkers” function in Seurat with default Wilcoxon rank sum test and filtered with the threshold of adjusted *P value* < 0.05 & |avg_log_2_FC|> 0.5. Thus, we conducted GSEA to identify the pathways that were induced or repressed in acinar cells during AP. The activity of multiple pathways in individual cells was also evaluated by single-cell GSVA and displayed by a UMAP plot. The Gene Ontology (GO) and Kyoto Encyclopedia of Genes and Genomes (KEGG) reference gene sets for GSEA and GSVA were acquired from MSigDB databases (https://www.gsea-msigdb.org/gsea/msigdb/).

### Pseudotime analysis of single cells

We generated the single-cell pseudotime trajectories with the Monocle3 package (1.2.9) in R [9]. The gene expression matrix in the scale of raw UMI counts derived from the Seurat processed data was used to create a CDS object with the new_cell_data_set() function. We performed procedures including normalization, FindVarialbeFeatures, ScaleData and RunPCA by the function preprocess_cds(). After removing the batch effect with the align_cds() function, we further reduced dimensionality with the UMAP method and generated a 2D visualization plot. In the next step, the pseudotime trajectories of acinar cells were constructed with WT acinar cells preset as the starting point. The pseudotime changes in the DEGs encoding transcription factors were visualized with a heatmap.

### Construction of PPI network and selection of hub genes

The common DEGs in acinar cells of GSE181276 and GSE188819 were introduced into the STRING database (https://www.string-db.org/) to obtain a complex PPI network. We visualized the PPI network with the Cytoscape software (version 3.8.2) and identified the top 18 genes in the MCC index as hub genes using cytohubba function in Cytoscape.

### Analysis of bulk RNA-seq data

The bulk RNA-seq dataset GSE194331 from human peripheral blood was used to research the biological features of AP patients in clinical terms. We identified the DEGs between SAP patients and healthy volunteers using DESeq2 R package (version 1.40.2) with the threshold of *P* value < 0.05 & |log_2_Foldchange|> 1 and take an intersection with the DEGs in acinar cells of GSE181276. The common DEGs were used for KEGG enrichment analysis with clusterProfiler R package (version 4.8.3).

## Results

### Overview of single-cell transcription profiling of the pancreatic tissue

**Fig. 1:**
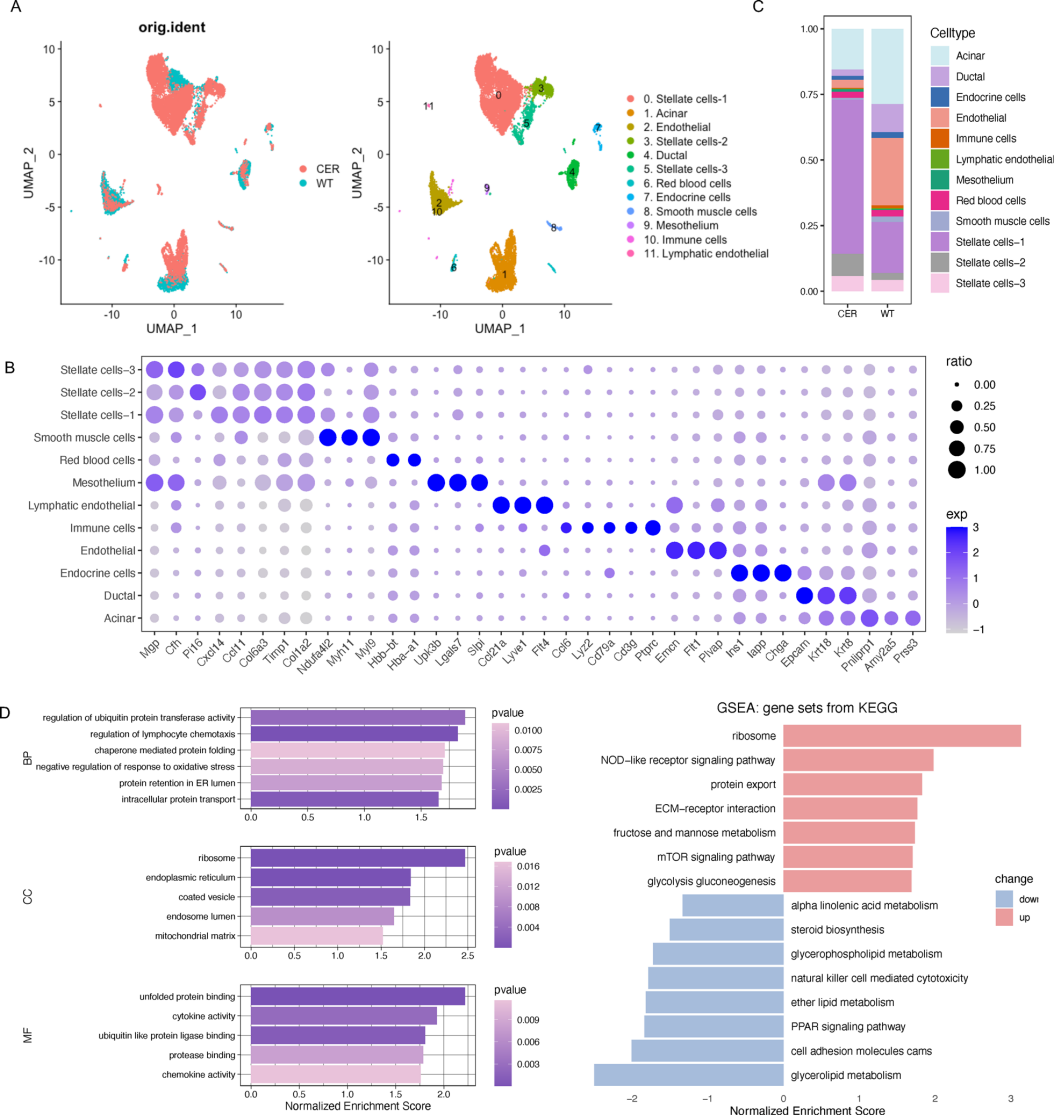
Overview of single-cell transcriptional profiling of the pancreatic tissue. **A** UMAP plot of cells derived from WT/CER samples (left) and the 12 identified clusters (right). **B** Dotplot visualization of marker genes for each cluster. The size of the dots indicates the ratio of cells expressing this gene, and the shade of color represents the mean transcription levels in the corresponding clusters. **C** Relative proportion of each cell type in WT/CER samples. **D** GO (left) and KEGG (right) enrichment analyses of DEGs between the CER and WT samples based on GSEA

After initial quality control and the removal of doublets, we obtained single-cell transcription data for 15,819 cells of GSE181276, including 5732 cells from mouse pancreatic tissues of wildtype (WT, untreated/control) group and 10,087 cells from mouse pancreatic tissues of cerulein (CER)-induced AP group. Based on the genetic map and typical markers, we divided these cells into 12 clusters, such as acinar cells, ductal cells, stellate cells, endothelial cells, and endocrine cells; these clusters were visualized with a UMAP plot (Fig. 1A). The dotplot shows the marker genes for each of these clusters (Fig. 1B). As demonstrated in Fig. 1C, the proportion of acinar cells in the AP sample was less than that in the WT sample, which might be related to programmed cell death [10]. Meanwhile, the proportion of stellate cells increased significantly in the AP sample, suggesting the important role of these cells in AP. The GSEA results revealed that pathways such as the “regulation of ubiquitin protein transferase activity”, “chaperone-mediated protein folding”, the “mTOR signaling pathway” and “glycolysis gluconeogenesis” were significantly activated (Fig. 1D). Studies have shown that mTOR signaling is related to the acinar-to-dendritic cell transition and the CD4^+^ T cell immune response in AP [11]. An additional file shows the overview of single-cell transcription profiling of the pancreas from GSE188819 [see Additional file 1: Fig. S1A–C]. In conclusion, we identified different cell types and their markers and performed GSEA in the pancreas from AP mice.

### scRNA-seq analysis revealed the alteration of transcription programs in acinar cells

**Fig. 2:**
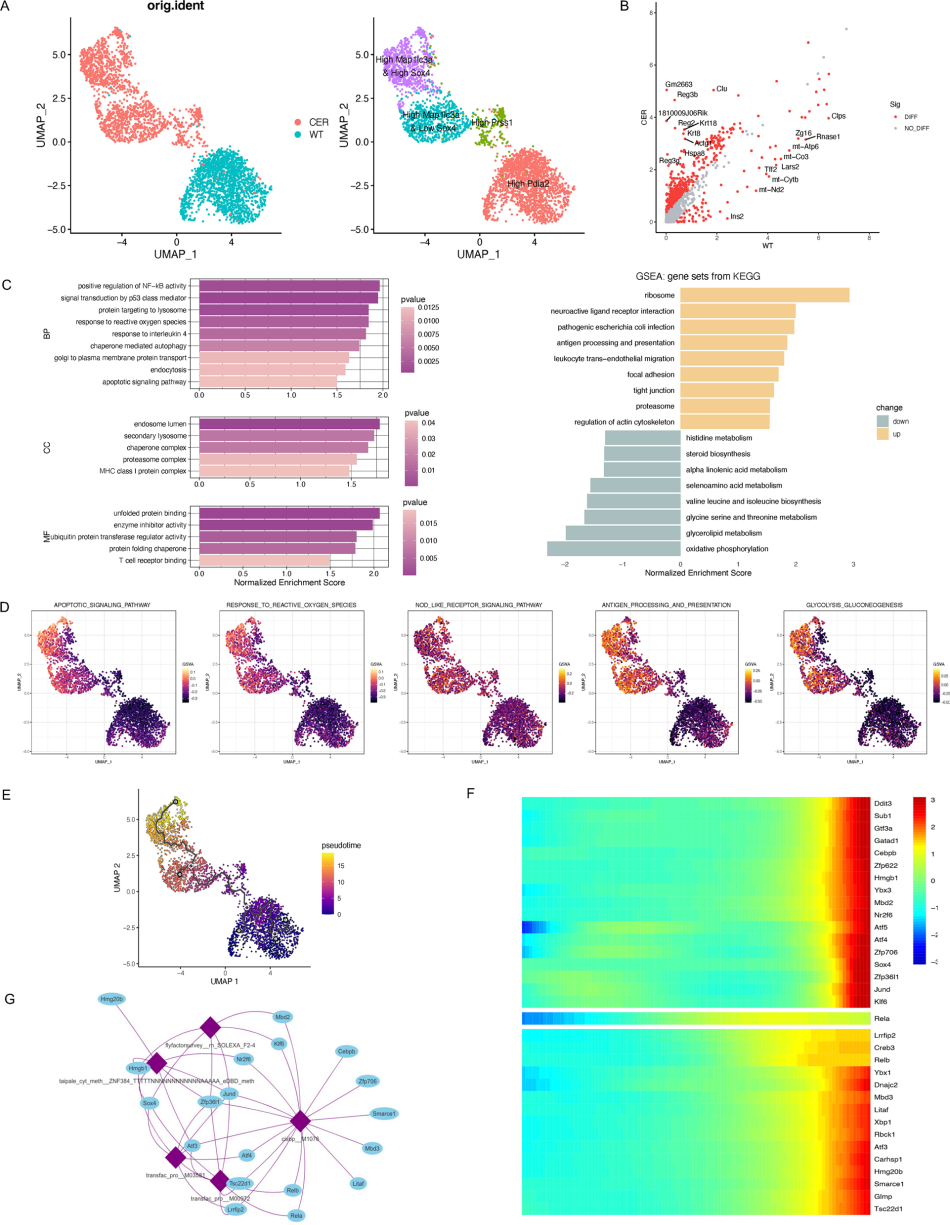
scRNA-seq analysis revealed the alteration of transcription programs in acinar cells. **A** UMAP plot of acinar derived from WT/CER samples (left) and subpopulations (right). **B** Scatter plot of DEGs (red points) in acinar cells showing the normalized expression level in CER (Y-axis) versus WT (X-axis) samples. The top 10 DEGs are highlighted by labels. **C** GO (left) and KEGG (right) enrichment analyses of DEGs in acinar cells based on GSEA. **D** UMAP plot visualizing the single-cell activity of specific pathways with GSVA scores. **E** Pseudotime trajectory of acinar cells in WT and CER samples. **F** Heatmap displaying the pseudotime changes of transcription factors (TFs) in acinar cells. **G** Binding motifs and expression regulatory network of the TFs in **F**

Acinar cells have been recognized as the site of onset of AP [3]. Thus, these cells were further extracted and divided into four subpopulations (Fig. 2A). Most cells from the WT sample highly expressed Pida2, while most cells from the AP sample belonged to the following other three subpopulations: high Prss1, high Map1lc3a & low Sox4, and high Map1lc3a & high Sox4 (Fig. 2A). The scatter plot shows the relative transcription levels of DEGs between the AP and WT samples, and the top 10 up- and downregulated genes are labelled (Fig. 2B). GSEA demonstrated that these DEGs were mainly enriched in pathways involving lysosomes, autophagy, ROS, endocytosis and apoptosis (Fig. 2C). According to single-cell GSVA scores, the activities of pathways including apoptosis, the response to ROS, the NOD-like receptor, antigen processing and presentation and glycolysis gluconeogenesis were significantly increased in the high Map1lc3a & low Sox4 cluster and the high Map1lc3a & high Sox4 cluster (Fig. 2D). We performed pseudotime trajectory analysis using monocle3, with WT acinar cells preset as the starting point. The high Map1lc3a & high Sox4 cluster was distributed in the late stage of the differentiation trajectory (Fig. 2E). The pseudotime changes in DEGs encoding transcription factors are shown in a heatmap (Fig. 2F). We further identified the binding motifs of these transcription factors and constructed an expression regulatory network (Fig. 2G). An additional file shows the altered transcription programs in acinar cells of GSE188819 [see Additional file 1: Fig. S1D–F]. In this part, we identified the subclusters and activated pathways of acinar cells in AP. The key transcription factors and regulatory network were also predicted.

### Acinar to ductal metaplasia (ADM) in AP

**Fig. 3:**
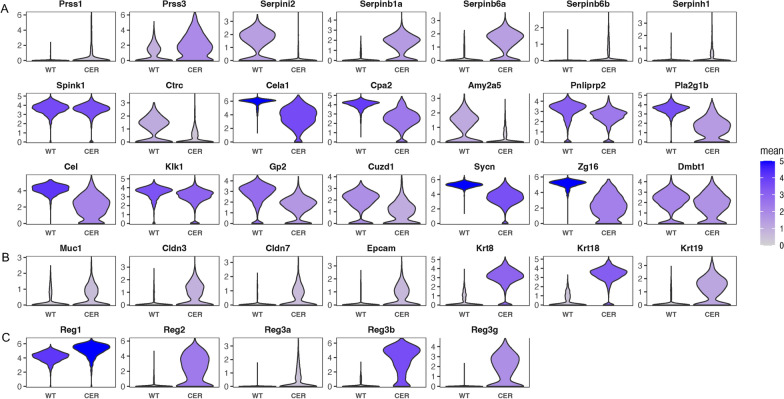
Acinar to ductal metaplasia (ADM) in AP. **A** Transcription levels of multiple enzymes and components of the zymogen granule membrane in acinar cells. **B** Transcription levels of ductal marker genes in acinar cells. **C** Transcription levels of regenerating family members in acinar cells

Acinar cells are plastic to some extent and can rapidly undergo a trans-differentiation process following inflammation or injury [12]. As shown, the expression of most genes encoding pancreatic digestive enzymes (Ctrc, Cela1, Amy2a5, Pnliprp2, Pla2g1b, Cel, Klk1) and components of zymogen granule membranes (Gp2, Cuzd1, Sycn, Zg16, Dmbt1) was downregulated in the AP sample, except for trypsinogen Prss1 and Prss3 (Fig. 3A; Additional file 2: Fig. S2A, B). Meanwhile, the transcription of ductal markers, including Muc1, Cldn3, Cldn7, Epcam, Krt8, Krt18 and Krt19, increased significantly (Fig. 3B; Additional file 2: Fig S2B). Interestingly, changes in serine protease inhibitors appeared to depend on their subcellular location. The expression of members secreted outside acinar cells (Serpini2) was downregulated. Others located in the cytoplasm or endoplasmic reticulum lumen, such as Serpinb1a, Serpinb6a, Serpinb6b and Serpinh1, were upregulated (Fig. 3A; Additional file 2: Fig. S2A). This may indicate acinar injury from self-digestion due to intracellular activation of trypsinogen. In addition, the regenerating family members, involved in cell proliferation and differentiation [13], also increased (Fig. 3C; Additional file 2: Fig. S2C). The loss of acinar markers and the increase of ductal markers revealed the ADM process of acinar cells in AP.

### Endocytosis and endosomal recycling were promoted

**Fig. 4:**
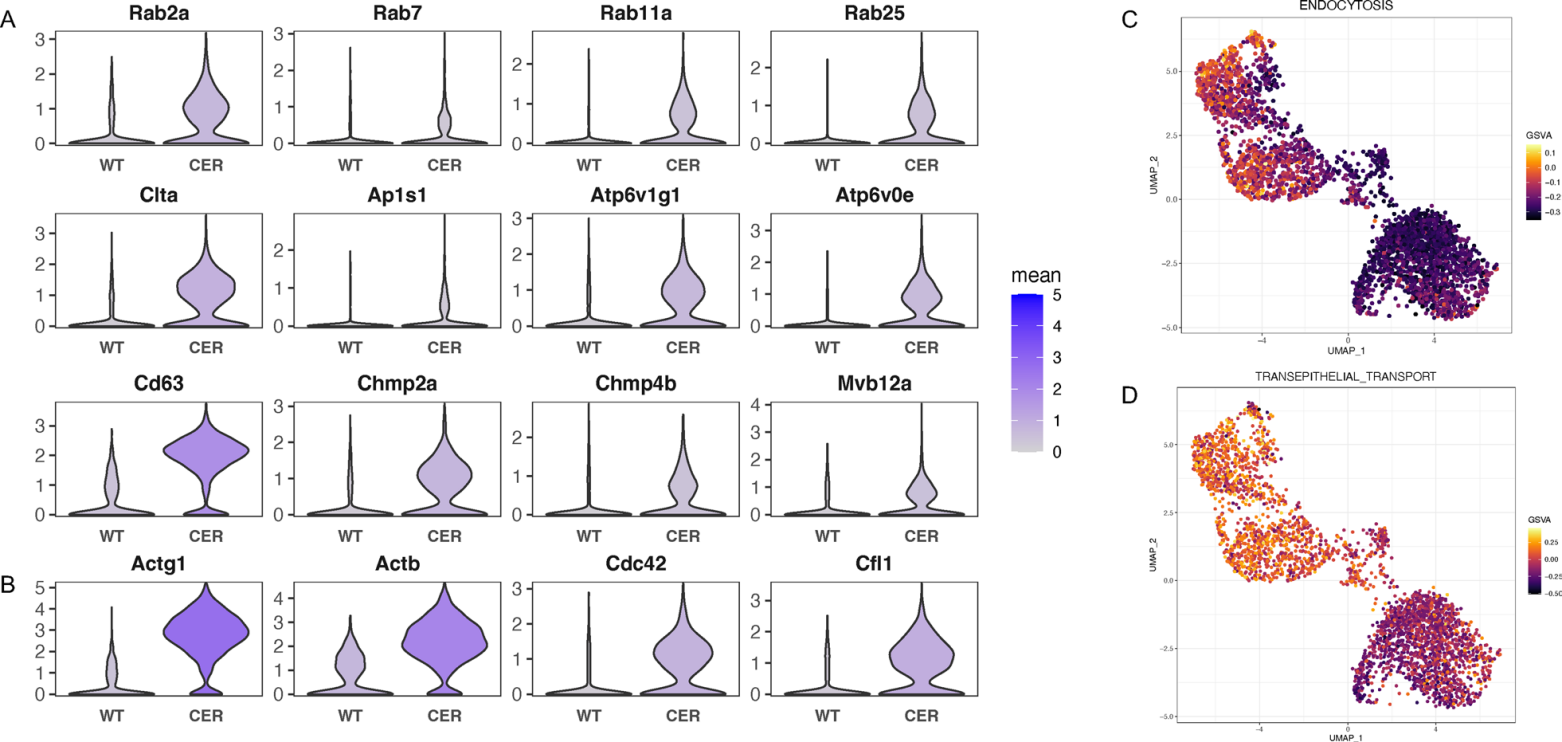
Endocytosis and endosomal recycling were promoted. **A** Transcription levels of endocytosis-, vesicular transportation- and endosomal recycling-associated genes. **B** Transcription levels of cytoskeleton-related genes. **C** UMAP plot depicting the single-cell activity of the “endocytosis” pathway with the GSVA score. **D** UMAP plot depicting the single-cell activity of the “trans-epithelial transport” pathway with the GSVA score

The critical function of the exocrine pancreas is to secrete digestive enzymes through exocytosis. At the same time, acinar cells undergo endocytosis and endosomal recycling. We found that the expression of markers of endocytosis and vesicular transportation (Rab2a, Rab7, Rab11a, Rab25, Clta, Ap1s1, Atp6v1g1, Atp6v0e) were increased in AP, as were the components of multivesicular bodies (Cd63, Chmp2a, Chmp4b, Mvb12a) and the transit depot of endosomal recycling (Fig. 4A; Additional file 3: Fig. S3A). Increased transcription of cytoskeleton-related genes (Actg1, Actb, Cdc42, Cfl1), which are essential for endocytosis, was also observed (Fig. 4B; Additional file 3: Fig S3B). Furthermore, the activity of the “endocytosis” and “trans-epithelial transport” pathways was remarkably elevated, particularly in the high Map1lc3a & low Sox4 and high Map1lc3a & high Sox4 clusters (Fig. 4C, D; Additional file 3: Fig. S3C, D). In conclusion, endocytosis and endosomal recycling were promoted in AP.

### Endoplasmic reticulum stress (ERS) was increased significantly

**Fig. 5:**
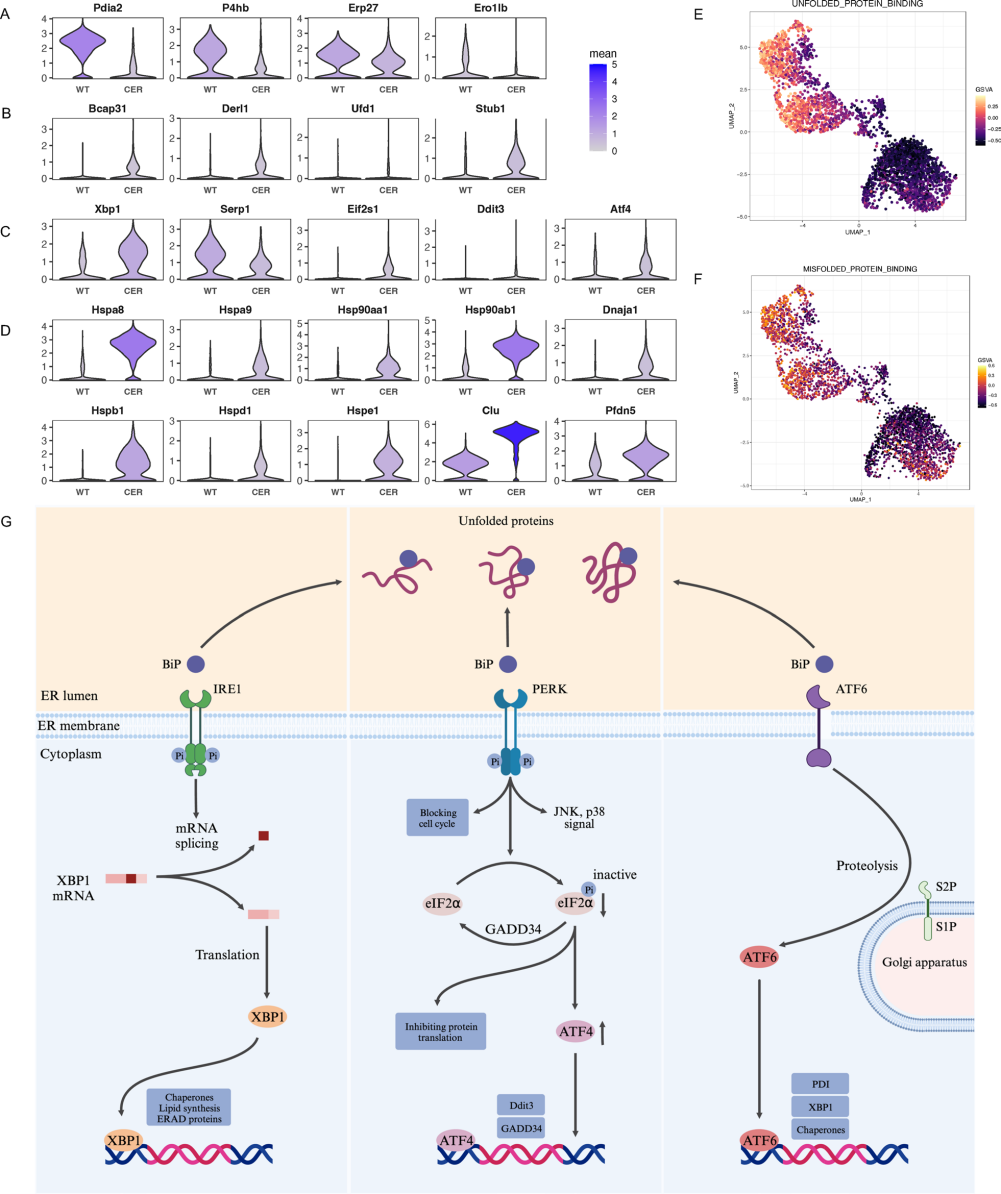
Endoplasmic reticulum stress was increased significantly. **A** Transcription levels of multiple genes involved in disulfide bond formation. **B** Transcription levels of genes involved in transportation across the ER membrane and the ubiquitin-mediated degradation of misfolded/unfolded proteins. **C** Transcription levels of ER stress marker genes. **D** Transcription levels of molecular chaperones that assist in protein folding. **E** UMAP plot depicting the single-cell activity of the “unfolded protein binding” pathway with the GSVA score. **F** UMAP plot depicting the single-cell activity of the “misfolded protein binding” pathway with the GSVA score. **G** Role of the unfolded protein response (UPR) in alleviating ERS

The endoplasmic reticulum (ER) is an intracellular factory for protein and lipid processing. The expression of multiple genes involved in disulfide bond formation (Pdia2, P4hb, Erp27, Ero1lb), which assist proteins in forming correct spatial conformation, was obviously downregulated in AP (Fig. 5A; Additional file 4: Fig. S4A). This downregulation may lead to the accumulation of misfolded/unfolded proteins and ERS (Fig. 5G). The ubiquitin‒proteasome system is one way to degrade misfolded/unfolded proteins in the ER; this system involves protein ubiquitylation and transportation from the ER into the cytoplasm. Increased transcription of related genes (Bcap31, Derl1, Ufd1, Stub1) reflected ERS in acinar cells (Fig. 5B; Additional file 4: Fig. S4B). We also found that the expression of key markers of ERS, including Xbp1, Serp1, Eif2s1, Ddit3, Atf4 (Fig. 5C; Additional file 4: Fig. S4C) and various molecular chaperones (Fig. 5D; Additional file 4: Fig S4D), was upregulated significantly. In addition, the GSVA scores of misfolded/unfolded protein binding pathways were higher in the AP group (Fig. 5E, F; Additional file 4: Fig. S4E, F). These results indicated increased ERS in acinar cells during AP.

### The ubiquitin‒proteasome pathway was activated

**Fig. 6:**
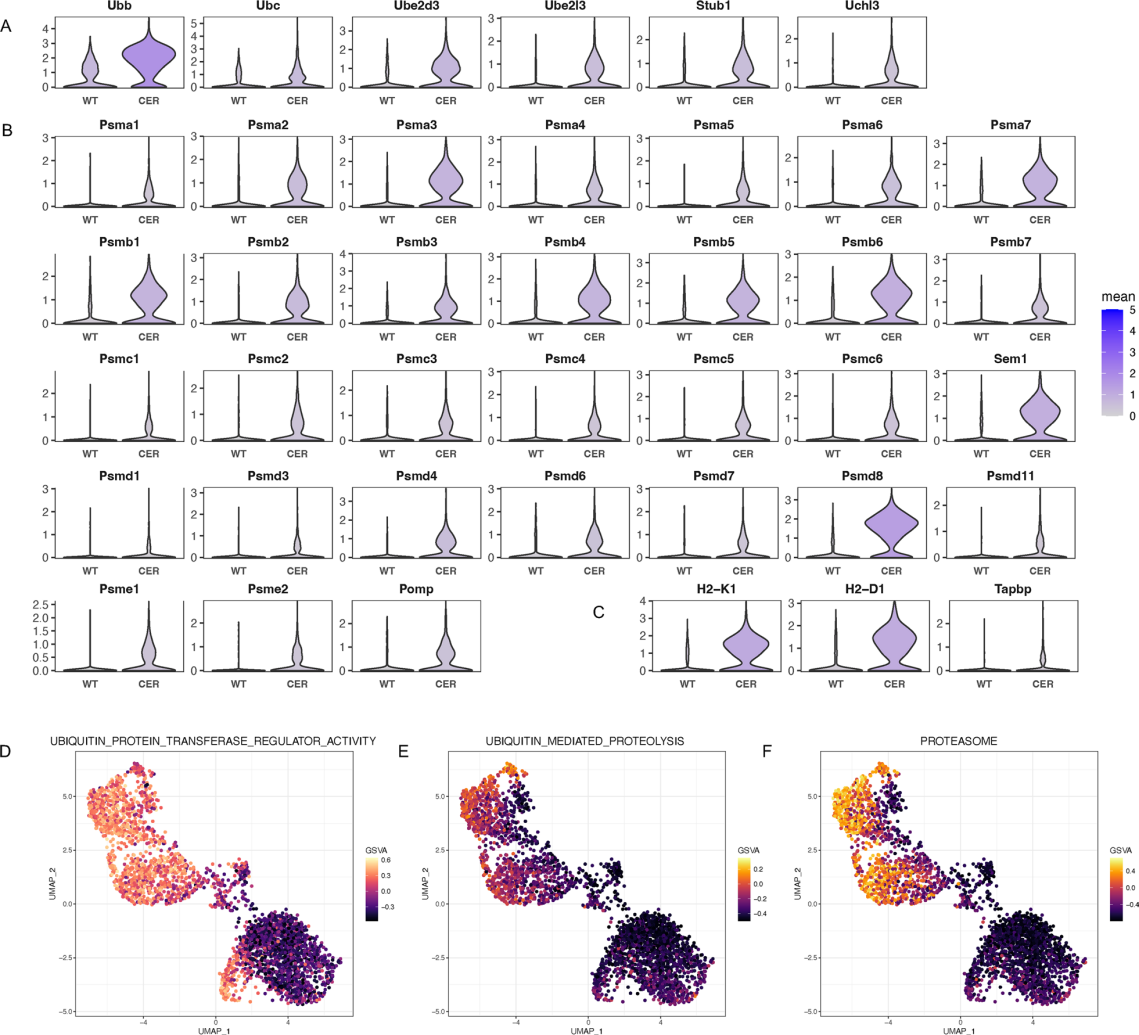
The ubiquitin–proteasome pathway was activated. **A** Transcription levels of ubiquitin and associated enzymes. **B** Transcription levels of proteasome components and Pomp. **C** Transcription levels of genes involved in the assembly of MHC-I and antigen peptide complexes. **D** UMAP plot depicting the single-cell activity of the “ubiquitin protein transferase regulator activity” pathway with the GSVA score. **E** UMAP plot depicting the single-cell activity of the “ubiquitin-mediated proteolysis” pathway with the GSVA score. **F** UMAP plot depicting the single-cell activity of the “proteasome” pathway with GSVA score

The ubiquitin‒proteasome system can degrade misfolded/unfolded proteins in the ER; this system is also known as the endoplasmic reticulum-associated degradation I pathway (ERAD-I) [14]. Single-cell data showed that the transcription levels of ubiquitin (Ubb, Ubc) and related enzymes (Ube2d3, Ube2l3, Stub1, Uchl3) were elevated in the acinar cells of the AP sample (Fig. 6A; Additional file 5: Fig. S5A). At the same time, the expression of subunits of the proteasome and Pomp, which promote proteasome assembly and maturation, was upregulated (Fig. 6B; Additional file 5: Fig. S5B). The ubiquitin‒proteasome system is also responsible for endogenous antigen processing. Processed antigen peptides need to cross the ER membrane with the help of the protein TAP (transporter associated with antigen processing) and then bind to MHC class I molecules [15]. The TAP binding protein (encoded by Tapbp) mediates the interaction between newly assembled MHC class I molecules and TAP [15]. We also found significantly increased transcription of MHC class I molecules and Tapbp (Fig. 6C; Additional file 5: Fig. S5C). Moreover, the results of single-cell GSVA (Fig. 6D–F; Additional file 5: Fig. S5D, E) also suggested that the ubiquitin proteasome pathway was activated.

### The transcription of autophagy‒lysosome pathway associated proteins was increased

**Fig. 7:**
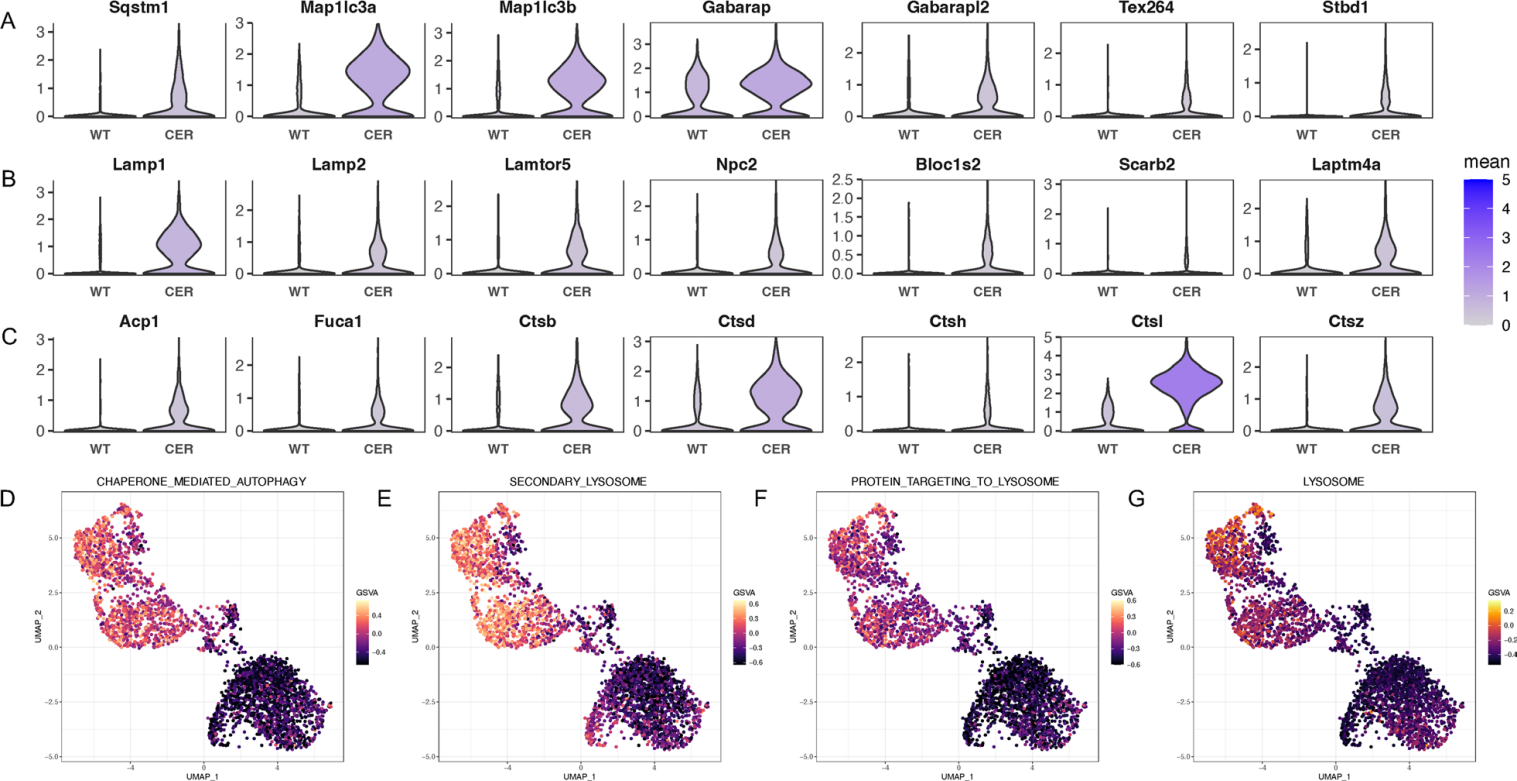
The transcription of autophagy–lysosome pathway associated proteins was increased. **A** Transcription levels of autophagy-related genes. **B** Transcription levels of lysosomal membrane components. **C** Transcription levels of lysosomal enzymes. **D** UMAP plot depicting the single-cell activity of the “chaperone-mediated autophagy” pathway with the GSVA score. **E** UMAP plot depicting the single-cell activity of the “secondary lysosome” pathway with the GSVA score. **F** UMAP plot depicting the single-cell activity of the “protein targeting to lysosome” pathway with the GSVA score. **G** UMAP plot depicting the single-cell activity of the “lysosome” pathway with GSVA score

The autophagy‒lysosome system, another EARD-II pathway for the degradation of misfolded/unfolded proteins in the ER [14], was also activated during AP. The transcription of autophagy-related genes (Sqstm1, Map1lc3a, Map1lc3b, Gabarap, Gabarapl2, Tex264, Stbd1) increased in the AP group (Fig. 7A; Additional file 6: Fig. S6A). Notably, Tex264 was identified as a major receptor of ER-phagy that remodels subdomains of the ER into autophagosomes, which is thought to be important for alleviating ERS [16]. The role of Tex264 in AP has not yet been reported. Meanwhile, the expression of components of the lysosomal membrane (Fig. 7B; Additional file 6: Fig. S6C) and lysosomal enzymes (Fig. 7C; Additional file 6: Fig. S6B) was upregulated. Additionally, increased activities of autophagy- and lysosome-related pathways were detected based on GSVA (Fig. 7D–G; Additional file 6: Fig. S6D–G). The autophagy-lysosome pathway may play a vital role in the pathogenesis of AP [17, 18].

### Integrated analysis of another scRNA-seq and bulk RNA-seq data

**Fig. 8:**
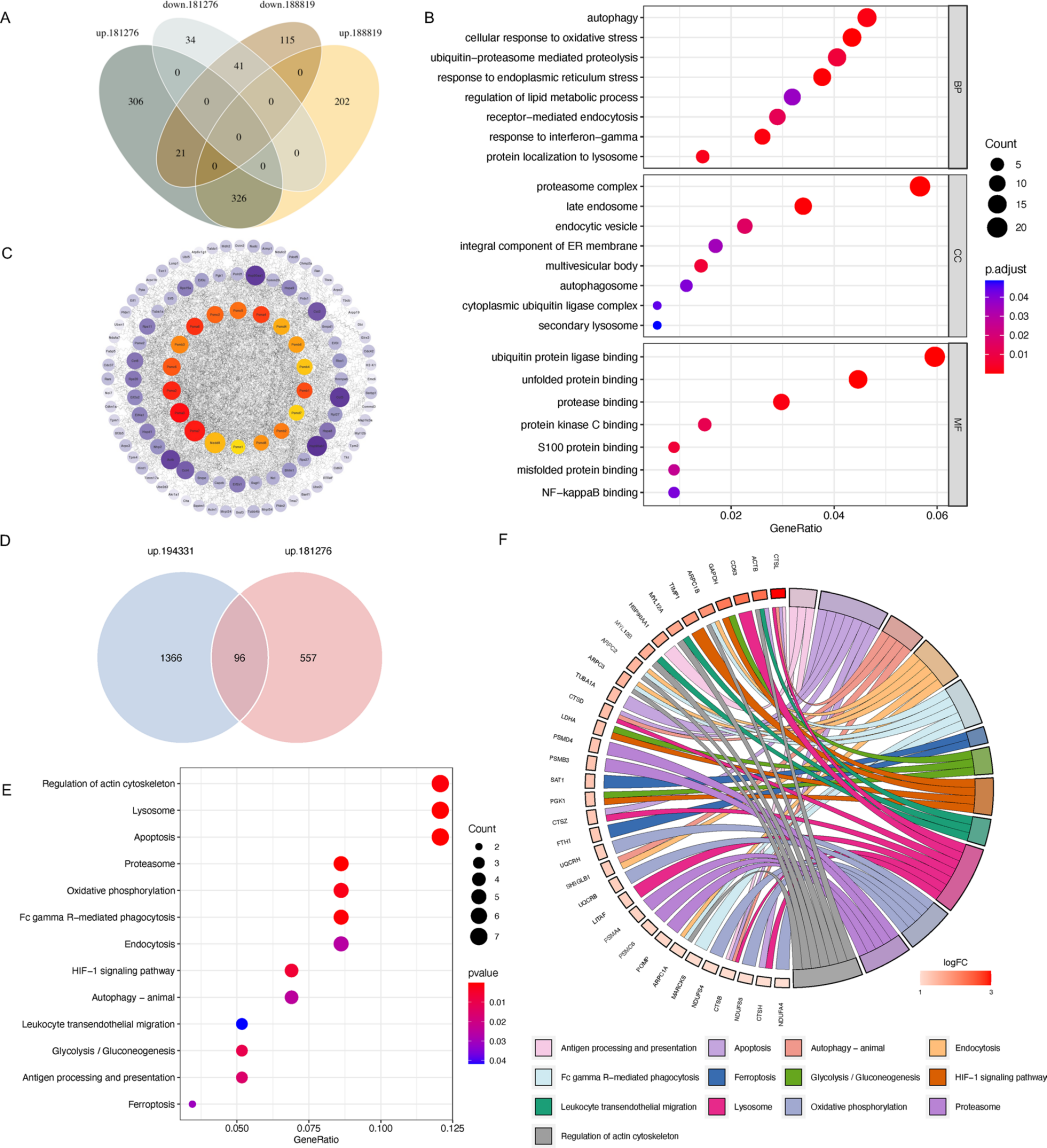
Integrated analysis of another scRNA-seq and bulk RNA-seq data. **A** Venn diagram indicating the common DEGs in the scRNA-seq datasets GSE181276 and GSE188819. **B** GO enrichment of common DEGs in the GSE181276 and GSE188819 datasets. **C** Protein‒protein interaction (PPI) network showing the common DEGs in **A**. **D** Venn diagram indicating the common upregulated DEGs in the scRNA-seq dataset GSE181276 and the bulk RNA-seq dataset GSE194331. **E** KEGG enrichment of the common upregulated DEGs in the GSE181276 and GSE194331 datasets. **F** Chord diagram demonstrating the enriched KEGG pathways in **E** and relevant upregulated genes

Another scRNA-seq dataset of mouse pancreas samples from GSE188819 and a bulk RNA-seq dataset of human peripheral blood samples from GSE194331 were analyzed. Common DEGs of acinar cells from GSE181276 and GSE188819 (Fig. 8A) were mainly enriched in pathways such as those involving autophagy, the lysosome, the proteasome, ERS and endocytosis (Fig. 8B). Moreover, the PPI network was generated by Cytoscape, and the top 18 genes of the maximum clique centrality (MCC) index were identified as hub genes and labeled with red dots (Fig. 8C). Interestingly, the hub genes all encoded components of the proteasome. We also analyzed human peripheral blood samples of 32 healthy volunteers and 10 SAP patients from GSE194331. Common DEGs between GSE181276 and GSE194331 (Fig. 8D) were similarly enriched in pathways involving endocytosis, the proteasome, the lysosome, autophagy and apoptosis (Fig. 8E, F). These results were consistent with the findings based on GSE181276 dataset and highlighted the importance of ERS, the ubiquitin‒proteasome pathway and the autophagy‒lysosome pathway in AP.

## Discussion

Acute pancreatitis is an acute abdominal disease that is common in clinical practice. Severe cases are often accompanied by systemic inflammation, as well as multiple organ failure, and patients often have a poor prognosis [19]. However, the pathogenesis of AP remains unclear. Acinar cells are commonly regarded as the initial site of AP onset [3], so we analyzed the transcriptional changes in acinar cells in the early stages of AP by integrating scRNA-seq and bulk RNA-seq data in this study.

In the single-cell sequencing data, we identified different cell clusters, including acinar cells, ductal cells, stellate cells, endocrine cells, endothelial cells, mesothelial cells, smooth muscle cells, red blood cells, and immune cells, as well as their representative markers. We compared the differences in each cell population in AP and WT samples. The proportions of stellate cells (Fig. 1B) and immune cells (Fig. 8B) was increased significantly in the AP group. Stellate cells are often thought to be associated with chronic pancreatitis. Recent research has shown that toxic Ca^2+^ signaling and NO in stellate cells can promote acinar injury and exacerbate inflammation in AP [20]. Immune disorder is a prominent feature of AP, including an early excessive immune response and late immunosuppression [21]. Xu et al. found that the mTOR-Myc axis could drive the acinar-to-DC transition, which promotes CD4^+^ T-cell differentiation to Th1 and Th17, thereby exacerbating the local inflammatory response [11].

Acinar cells were extracted and divided into four subgroups by unsupervised clustering method: high Pdia2, high Prss1, high Map1lc3a & low Sox4 and high Map1lc3a & high Sox4. Most cells from the WT sample belonged to the high Pida2 cluster. The reduction of multiple enzymes catalyzing protein folding [22] including Pdia2, Pdia4 and P4hb, may be the reason for over-accumulated misfolded/unfolded proteins and ERS in acinar cells. Microtubule-associated protein 1 light chain 3 (LC3) encoded by Map1lc3a and Map1lc3b play a vital role in autophagy substrate selection and autophagosome biogenesis [23]. A recent study has revealed that transgenic expression of GFP-LC3 disturbed autophagy flux and increased the formation of autophagy vacuoles in acinar cells [24]. SOX4 functioned in promoting cell proliferation [25], pancreatic development [26] and epithelial-mesenchymal transition [27], which has not been reported in acute pancreatitis.

Acinar cells rapidly undergo a trans-differentiation process stimulated by inflammation, which is called ADM [12]. In our study, at the early stage of AP, the transcription levels of most digestive enzymes and components of the zymogen granule membrane in acinar cells greatly decreased, while that of ductal epithelial-associated markers increased. This may be a mechanism by which acinar cells protect against self-digestion. However, surprisingly, the transcription levels of trypsinogens, such as Prss1 and Prss3, significantly increased. Interestingly, changes in genes encoding serine protease inhibitors appeared to be related to their subcellular location. The expression of serine protease inhibitors secreted outside the cell was downregulated, in contrast to other members located in the cytoplasm or endoplasmic reticulum lumen; these other members are thought to protect cells from leaking lysosomal enzymes or elastase secreted by neutrophils [28].

**Fig. 9:**
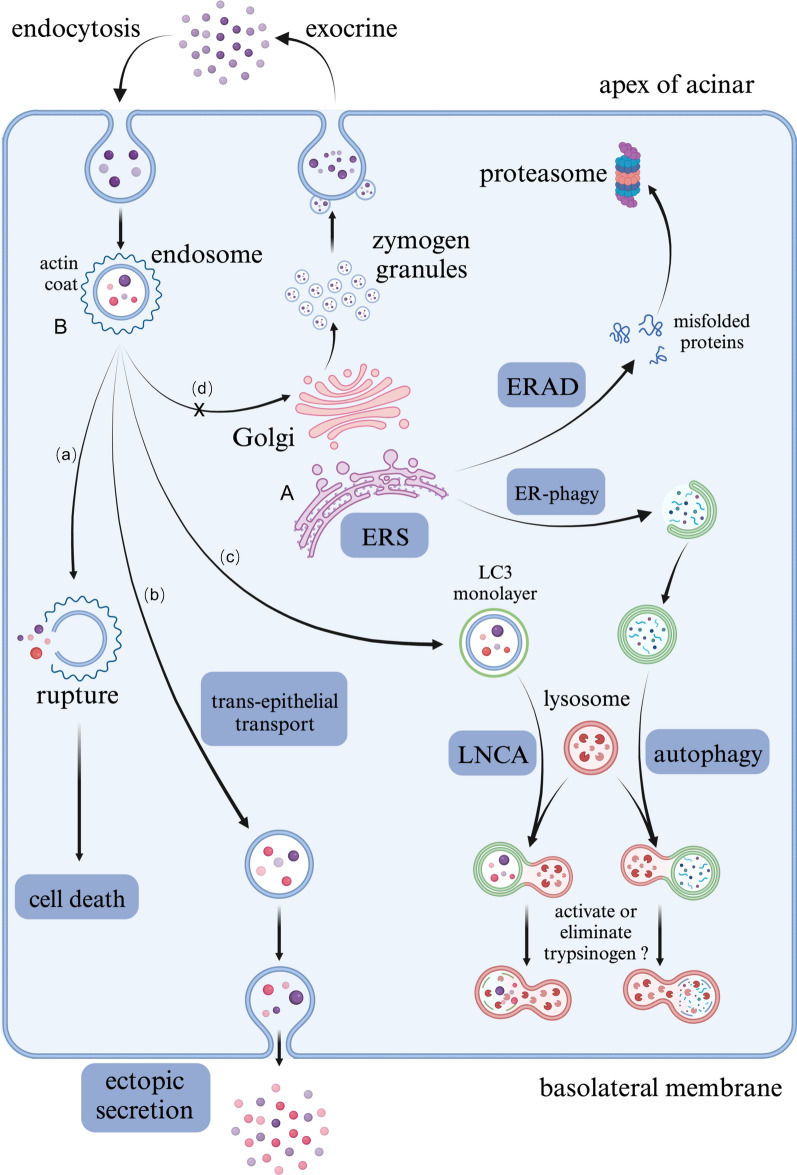
Biological characteristics of acinar cells involved in ERS in AP. **A** ERS signaling was significantly activated in acinar cells due to excessive unfolded/misfolded proteins, which could be degraded by ERAD pathways including ubiquitin–proteasome system and autophagy-lysosome system. **B** The fate of endocytic vesicles containing mixed enzymes and zymogens: **a** ruptured when losing the protection by actin coat, which led to cell death; **b** underwent trans-epithelial transport of long distance then secreted outside of cells ectopically; **c** being wrapped by monolayer LC3 and degraded by LNCA; **d** recirculated into secretory compartments, which was blocked during AP

We found that acinar cells showed significantly increased endocytosis and transepithelial transport in AP. Trypsinogen and lysosomal enzymes can be simultaneously engulfed in endocytic vesicles (EVs) [29], and the lysosomal enzyme cathepsin B is capable of activating trypsinogen [30]. The fate of these zymogen-enriched EVs deserves our attention (Fig. 9). Michael et al. demonstrated that F-actin wrapped and protected EVs from rupture, which was lethal to acinar cells [29]. As described by MM Lerch and his colleagues, during AP, these EVs are transported to lysosomes with blocked recirculation to the secretory compartment [31]. In addition, consistent with the increase in transepithelial transport, some EVs migrated from the apex of acinar cells to the basolateral membrane [29]. Alcohol or cholecystokinin (CCK) stimulated acinar cells increase basolateral exocytosis, leading to the ectopic release of trypsin [32]. LAP-like noncanonical autophagy (LNCA) is another outlet of these EVs, in which the F-actin shell is replaced by a monolayer of LC3 membrane [33]. Notably, LNCA took place only 10–20 min after CCK stimulation [33], indicating its potential to activate trypsinogen at an early stage. Trypsinogen activation has been detected in LC3-coated EVs [34]. However, considering the early decrease in trypsin activity in AP [35], LNCA may be responsible for trypsinogen degradation instead of activation.

Acinar cells have a large ER to accommodate the function of synthesizing digestive enzymes. In some pathological conditions, excessive unfolded or misfolded proteins accumulated in the ER and can induce ERS [36], which in turn activates NF-κB and programmed cell death pathways in acinar cells during AP [37]. Our analysis indicated increased ERS in acinar cells during AP, accompanied by an increase in molecular chaperones, including members of the Hsp10, Hsp20, Hsp60, Hsp70 and Hsp90 families. This increase may be a result of the decreased levels of disulfide isomerase and the inability of nascent proteins to form correct disulfide bonds. The main function of Hsp70 family members is to alleviate ERS by assisting in the proper folding of nascent polypeptides, as well as targeting misfolded proteins for degredation [38]. Several independent studies have confirmed that Hsp70 induced by thermal or nonthermal stress can prevent cerulein-induced AP by inhibiting trypsinogen and NF-κB activation [39–41].

There are two endoplasmic reticulum-associated degradation (ERAD) pathways for unfolded/misfolded proteins that accumulate in the ER, the ubiquitin‒proteasome system (ERAD-I) and the autophagy‒lysosome system (ERAD-II) [14]. We found that both of them were significantly activated in AP. Theoretically, the ubiquitin‒proteasome system contributes to attenuating ER pressure and maintaining acinar homeostasis. However, recent studies have shown that proteasome inhibitors protect against experimental acute pancreatitis by inhibiting the NF-κB pathway [42, 43]. The degradation products of the proteasome are usually short peptides 4–25 amino acids in length. However, in some cases, functional molecules are also produced, such as NF-κB and the yeast proteins SPT23 and MGA2 [44]. Whether trypsinogen can be activated through proteasome-dependent processing has not been determined.

Autophagy is another important degradation pathway that includes macroautophagy, microautophagy, and chaperone-mediated autophagy [45]; the role of autophagy in AP is somewhat controversial. As mentioned above, the lysosomal enzyme cathepsin B activates trypsinogen [30]. It is thought that autophagy mediates the colocalization of zymogen with lysosomal enzymes and abnormal activation [46]. Reduced severity of AP and trypsinogen activation in Atg5 knockout mice was reported by Hashimoto and colleagues [46]. However, in another study, knocking out Atg5 in the pancreas of mice caused spontaneous pancreatitis [47], similar to knocking out Atg7 [17]. Thus, some other scholars support the idea that autophagy plays a protective role by eliminating zymogens. Daniel et al. identified a selective autophagy, zymophagy, mediated by VMP1-USP9X-p62 in experimental acute pancreatitis; zymophagy can prevent cellular damage by degrading zymogen particles [48].

Mareninova et al. revealed that impaired autophagy resulted in trypsinogen activation and the vacuolization of acinar cells [49]. Multiple studies have demonstrated that lysosomal dysfunction impedes autophagic flux and exacerbates susceptibility to AP. Lysosome-associated membrane protein (LAMP) is an important component of lysosomes, and LAMP-2-deficient mice can develop spontaneous pancreatitis [50]. The transcription factor TFEB is responsible for regulating lysosomal biogenesis and the transcription of several genes involved in autophagy [51]. In cerulein-induced AP, acinar-specific TFEB knockout mice exhibited insufficient autophagy and more severe inflammation [52]. Gnptab encodes a key enzyme involved in the generation of M6P residues in newly synthesized lysosomal enzymes, which is important for their translocation to prelysosomes [53]. Gnptab−/− mice developed spontaneous pancreatitis, accompanied by the accumulation of nondegradative autolysosomes and increased trypsinogen activation [54]. The absence of CI-MPR (cation-independent mannose-6-phosphate receptor) causes the misdistribution of cathepsin B to the secretory compartment without inducing spontaneous trypsinogen activation [55]. However, during cerulein-induced pancreatitis, trypsinogen activation in CI-MPR-deficient mice was approximately 40% higher than that in wild-type mice [55].

In conclusion, we identified the cell types in the pancreas of AP mice and analyzed the transcriptional changes in acinar cells by single-cell transcriptome analysis. We demonstrated the characteristics of acinar cells in the early stage of AP, including ADM, increased endocytosis and transepithelial transport, ERS, and activation of the ubiquitin‒proteasome and autophagy‒lysosome pathways, among which ERS may play a central role (Fig. 9). In addition, a series of experiments should be conducted to clarify the regulatory mechanism of ERS in the pathogenesis of AP, including acinar cell specific knockout or overexpression of Xbp1, Pdia2 and P4hb, which will provide new ideas and strategies for developing clinical intervention and treatment methods.

### Supplementary Information

**Additional file 1: Figure S1.** Overview of single-cell transcription profiling of the pancreatic tissue from GSE188819.** A** UMAP plot of cells derived from WT/CER samples (left) and the 13 identified clusters (right).** B** Relative proportion of each cell type in WT/CER samples.** C** Dotplot displaying the marker genes in different types of cells. The size of the dots indicates the ratio of cells expressing this marker gene, and the shade of color represents the mean expression levels in the corresponding cell cluster.** D **UMAP plot of acinar cells from WT/CER samples (left) and the 4 identified clusters (right).** E **Pseudotime trajectory showing the dynamics of acinar cells from WT and CER samples using the Monocle3 tool.** F **Scatter plot of DEGs (red points) in acinar tissue showing the normalized expression levels in CER (Y-axis) versus WT (X-axis) samples. The top 10 DEGs are indicated by labels.

**Additional file 2: Figure S2. **Acinar to ductal metaplasia (ADM) in AP. **A** Transcription levels of multiple enzymes and components in the zymogen granule membrane in acinar cells. **B** Transcription levels of ductal marker genes in acinar cells. **C **Transcription levels of regenerating family members in acinar cells.

**Additional file 3: Figure S3. **Endocytosis and endosomal recycling were promoted.** A** Transcription levels of endocytosis-, vesicular transportation- and endosomal recycling-associated genes. **B **Transcription levels of cytoskeleton-related genes. **C **UMAP plot depicting the single-cell activity of the “endocytosis” pathway with the GSVA score. **D** UMAP plot depicting the single-cell activity of the “trans-epithelial transport” pathway with the GSVA score.

**Additional file 4: Figure S4. **Endoplasmic reticulum stress was increased significantly**. A **Transcription levels of multiple genes involved in disulfide bond formation. **B** Transcription levels of genes involved in transportation across the ER membrane and the ubiquitin-mediated degradation of misfolded/unfolded proteins. **C **Transcription levels of endoplasmic reticulum stress marker genes. **D **Transcription levels of molecular chaperones that assist in protein folding. **E **UMAP plot depicting the single-cell activity of the “unfolded protein binding” pathway with the GSVA score. **F **UMAP plot depicting the single-cell activity of the “misfolded protein binding” pathway with the GSVA score.

**Additional file 5: Figure S5: **The ubiquitin‒proteasome pathway was activated.** A** Transcription levels of ubiquitin and associated enzymes. **B **Transcription levels of proteasome components and Pomp. **C** Transcription levels of genes involved in the assembly of MHC-I and the antigen peptide complexes. **D** UMAP plot depicting the single-cell activity of the “ubiquitin-mediated proteolysis” pathway with the GSVA score. **E** UMAP plot depicting the single-cell activity of the “proteasome” pathway with the GSVA score.

**Additional file 6: Figure S6. **The transcription of autophagy‒lysosome pathway associated proteins was increased. **A** Transcription levels of autophagy-related genes. **B **Transcription levels of lysosomal membrane components. **C** Transcription levels of lysosomal enzymes. **D** UMAP plot depicting the single-cell activity of the “chaperone-mediated autophagy” pathway with the GSVA score. **E** UMAP plot depicting the single-cell activity of the “secondary lysosome” pathway with the GSVA score. **F** UMAP plot depicting the single-cell activity of the “protein targeting to lysosome” pathway with the GSVA score. **G** UMAP plot depicting the single-cell activity of the “lysosome” pathway with GSVA score.

## Data Availability

The GSE181276, GSE188819 and GSE194331 datasets were downloaded from the GEO database (https://www.ncbi.nlm.nih.gov/geo/). The data that support the findings of this study are available from the corresponding author.

## Abbreviations

AP: Acute pancreatitis
GSEA: Gene sets enrichment analysis
GSVA: Gene sets variation analysis
PPI: Protein–protein interaction
ADM: Acinar to ductal metaplasia
ERS: Endoplasmic reticulum stress
ERAD: ER-associated degradation
SAP: Severe acute pancreatitis
WT: Wildtype
scRNA-seq: Single-cell RNA sequencing
bulk RNA-seq: Bulk RNA sequencing
HVGs: Highly variable genes
PCA: Principal component analysis
UMAP: Uniform manifold approximation and projection
DEGs: Differentially expressed genes
GO: Gene Ontology
KEGG: Kyoto Encyclopedia of Genes and Genomes
CER: Cerulein
ER: Endoplasmic reticulum
MCC: Maximum clique centrality
LC3: Microtubule-associated protein 1 light chain 3
EVs: Endocytic vesicles
LNCA: LAP-like noncanonical autophagy
CCK: Cholecystokinin
LAMP: Lysosome-associated membrane protein
CI-MPR: Cation-independent mannose-6-phosphate receptor
