# Supplementary figures and images for: The integration of single-cell and bulk RNA-seq atlas reveals ERS-mediated acinar cell damage in acute pancreatitis
Source: J Transl Med. 2024 Apr 11;22:346. doi: 10.1186/s12967-024-05156-0 (PMC11010368; doi:10.1186/s12967-024-05156-0)

A

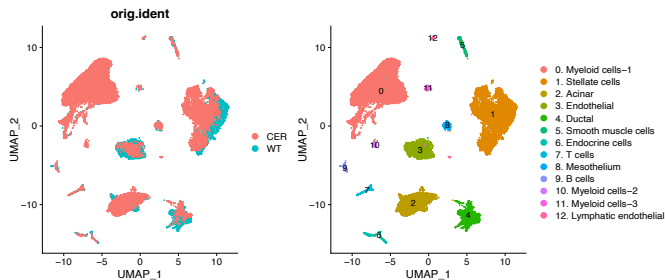

B

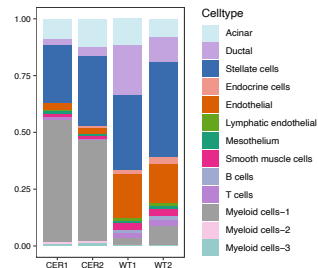

C

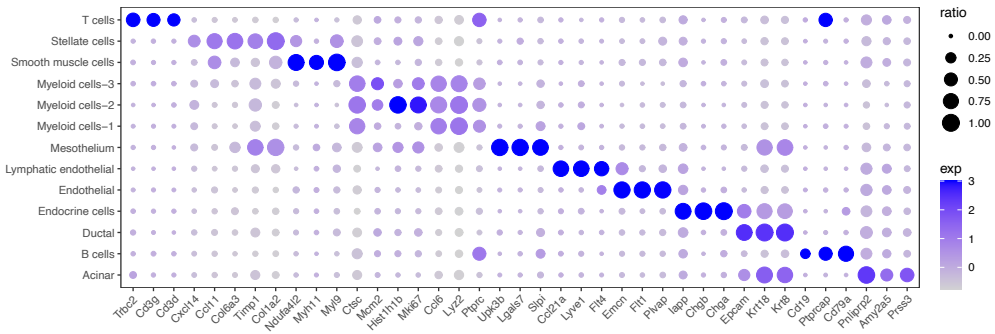

D

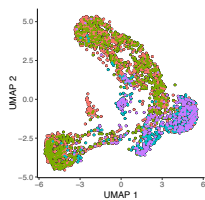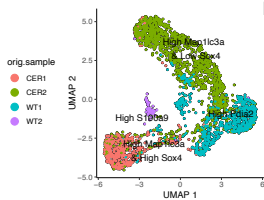

E

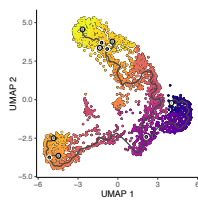

F

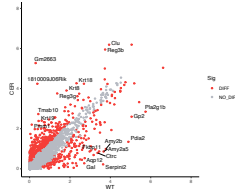

Supplement: Supplementary file 1 — Additional file 1: Figure S1. Overview of single-cell transcription profiling of the pancreatic tissue from GSE188819. A UMAP plot of cells derived from WT/CER samples (left) and the 13 identified clusters (right). B Relative proportion of each cell type in WT/CER samples. C Dotplot displaying the marker genes in different types of cells. The size of the dots indicates the ratio of cells expressing this marker gene, and the shade of color represents the mean expression levels in the corresponding cell cluster. D UMAP plot of acinar cells from WT/CER samples (left) and the 4 identified clusters (right). E Pseudotime trajectory showing the dynamics of acinar cells from WT and CER samples using the Monocle3 tool. F Scatter plot of DEGs (red points) in acinar tissue showing the normalized expression levels in CER (Y-axis) versus WT (X-axis) samples. The top 10 DEGs are indicated by labels. [file 12967_2024_5156_MOESM1_ESM.pdf]

A

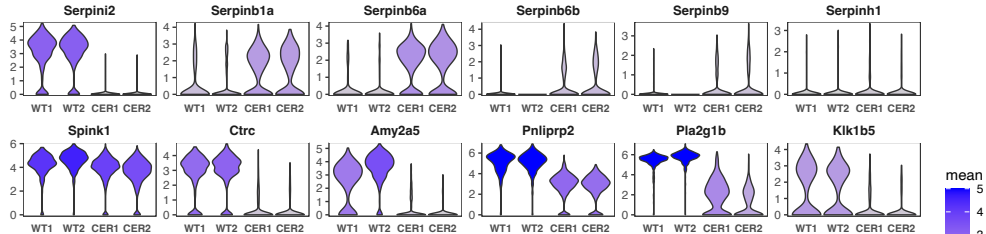

B

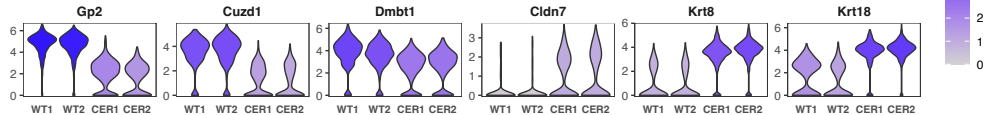

C

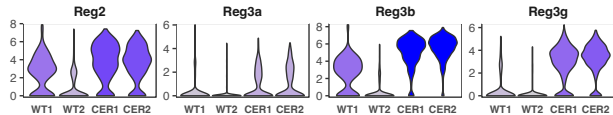

Supplement: Supplementary file 2 — Additional file 2: Figure S2. Acinar to ductal metaplasia (ADM) in AP. A Transcription levels of multiple enzymes and components in the zymogen granule membrane in acinar cells. B Transcription levels of ductal marker genes in acinar cells. C Transcription levels of regenerating family members in acinar cells. [file 12967_2024_5156_MOESM2_ESM.pdf]

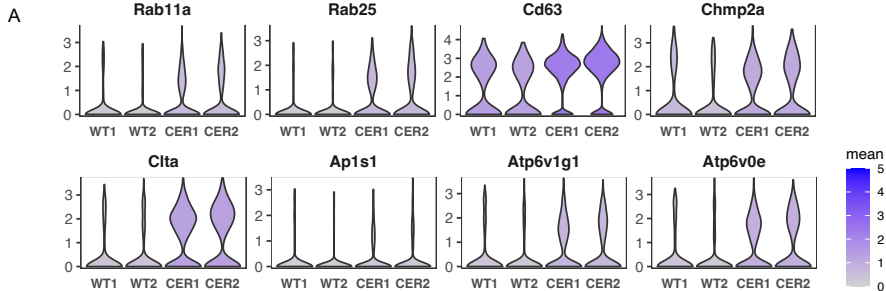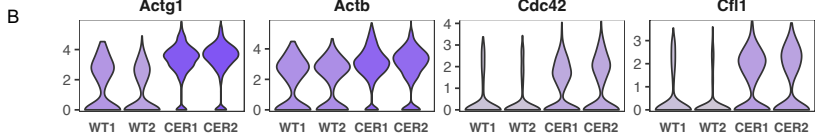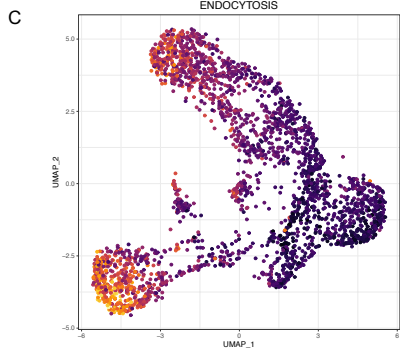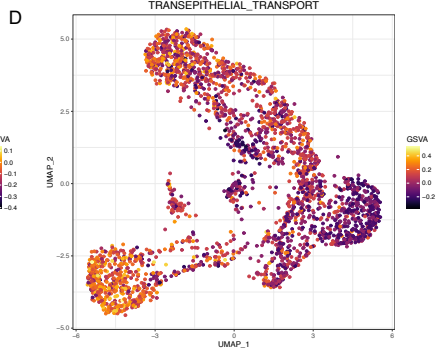

Supplement: Supplementary file 3 — Additional file 3: Figure S3. Endocytosis and endosomal recycling were promoted. A Transcription levels of endocytosis-, vesicular transportation- and endosomal recycling-associated genes. B Transcription levels of cytoskeleton-related genes. C UMAP plot depicting the single-cell activity of the “endocytosis” pathway with the GSVA score. D UMAP plot depicting the single-cell activity of the “trans-epithelial transport” pathway with the GSVA score. [file 12967_2024_5156_MOESM3_ESM.pdf]

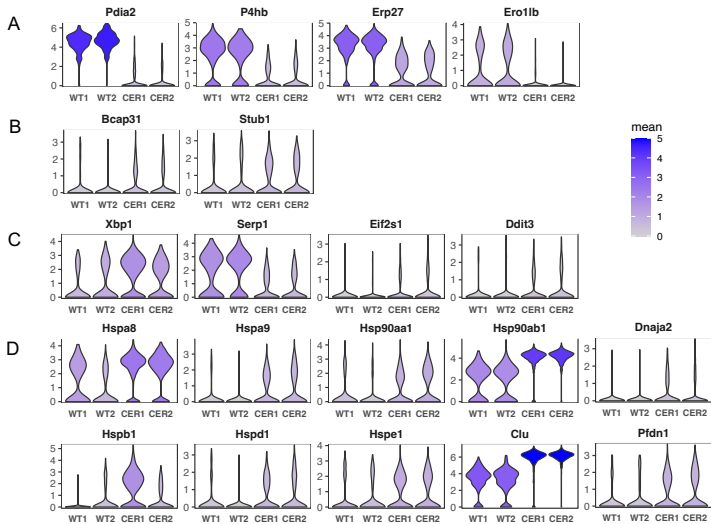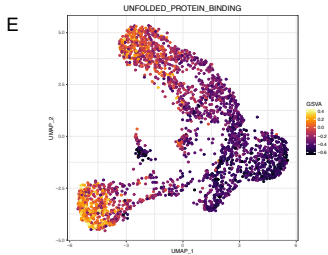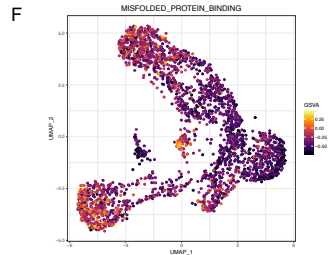

Supplement: Supplementary file 4 — Additional file 4: Figure S4. Endoplasmic reticulum stress was increased significantly. A Transcription levels of multiple genes involved in disulfide bond formation. B Transcription levels of genes involved in transportation across the ER membrane and the ubiquitin-mediated degradation of misfolded/unfolded proteins. C Transcription levels of endoplasmic reticulum stress marker genes. D Transcription levels of molecular chaperones that assist in protein folding. E UMAP plot depicting the single-cell activity of the “unfolded protein binding” pathway with the GSVA score. F UMAP plot depicting the single-cell activity of the “misfolded protein binding” pathway with the GSVA score. [file 12967_2024_5156_MOESM4_ESM.pdf]

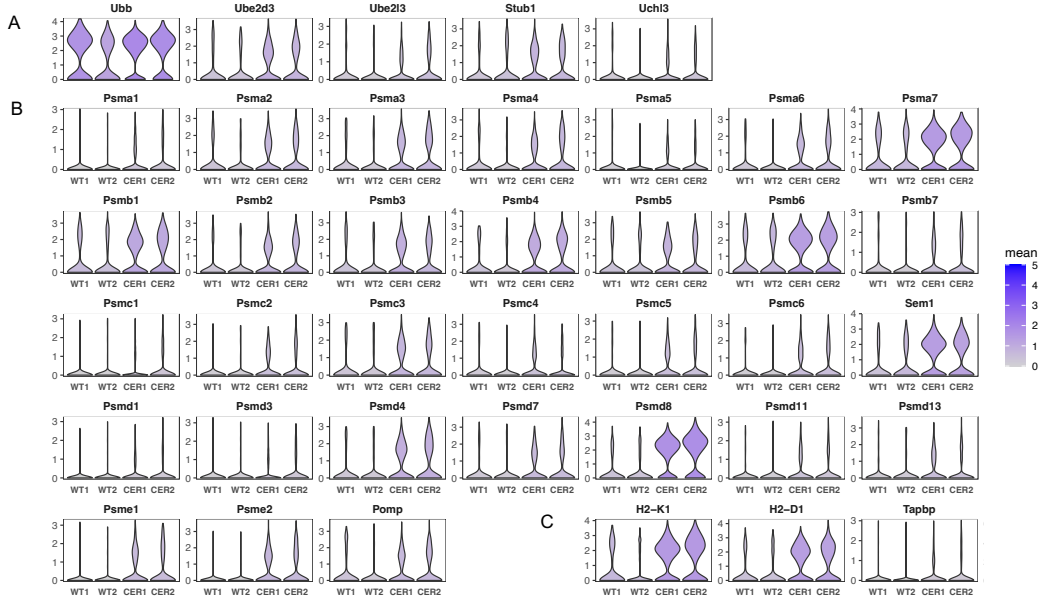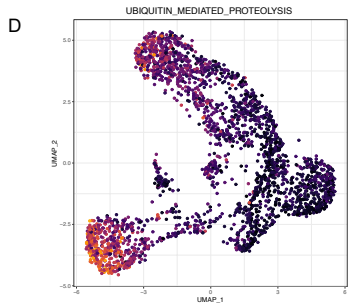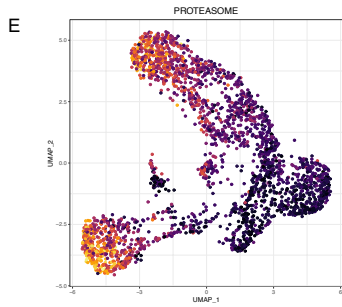

Supplement: Supplementary file 5 — Additional file 5: Figure S5: The ubiquitin‒proteasome pathway was activated. A Transcription levels of ubiquitin and associated enzymes. B Transcription levels of proteasome components and Pomp. C Transcription levels of genes involved in the assembly of MHC-I and the antigen peptide complexes. D UMAP plot depicting the single-cell activity of the “ubiquitin-mediated proteolysis” pathway with the GSVA score. E UMAP plot depicting the single-cell activity of the “proteasome” pathway with the GSVA score. [file 12967_2024_5156_MOESM5_ESM.pdf]

A

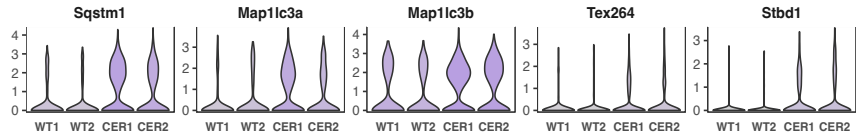

B

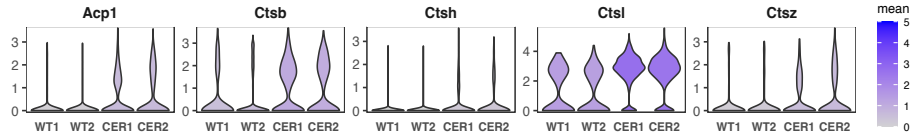

C

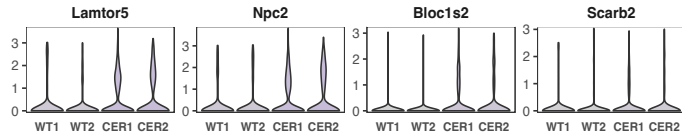

D

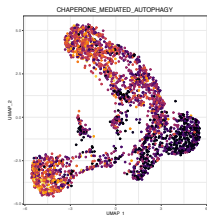

E

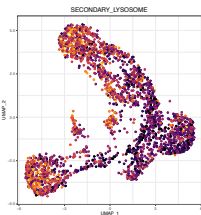

F

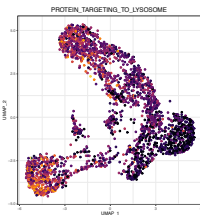

G

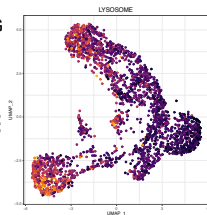

Supplement: Supplementary file 6 — Additional file 6: Figure S6. The transcription of autophagy‒lysosome pathway associated proteins was increased. A Transcription levels of autophagy-related genes. B Transcription levels of lysosomal membrane components. C Transcription levels of lysosomal enzymes. D UMAP plot depicting the single-cell activity of the “chaperone-mediated autophagy” pathway with the GSVA score. E UMAP plot depicting the single-cell activity of the “secondary lysosome” pathway with the GSVA score. F UMAP plot depicting the single-cell activity of the “protein targeting to lysosome” pathway with the GSVA score. G UMAP plot depicting the single-cell activity of the “lysosome” pathway with GSVA score. [file 12967_2024_5156_MOESM6_ESM.pdf]
